# Efficacy and Tolerability of the Fixed Combinations Latanoprost/Timolol versus Dorzolamide/Timolol in Patients with Elevated Intraocular Pressure: A Meta-Analysis of Randomized Controlled Trials

**DOI:** 10.1371/journal.pone.0083606

**Published:** 2013-12-11

**Authors:** Miao He, Wei Wang, Wenyong Huang

**Affiliations:** Zhongshan Ophthalmic Center, State Key Laboratory of Ophthalmology, Sun Yat-Sen University, Guangzhou, People’s Republic of China; Casey Eye Institute, United States of America

## Abstract

**Objective:**

To evaluate the efficacy and tolerability of the fixed combination of Latanoprost/Timolol versus Dorzolamide/Timolol in the treatment of patients with elevated intraocular pressure (IOP).

**Methods:**

A comprehensive literature meta-analysis was performed according to the Cochrane Collaboration methodology to identify randomized clinical trials comparing latanoprost/timolol FC (FCLT) with dorzolamide/timolol (FCDT) in patients with elevated IOP. The efficacy estimates were measured by the weight mean difference (WMD) for the IOP reduction (IOPR) from baseline to end point, including the diurnal mean IOPR, 8 AM IOPR, 12 PM IOPR, and 4 PM IOPR. The tolerability estimates were measured by RR for adverse events. All outcomes were reported with a 95% confidence interval (CI). The data were synthesized by Stata 12.0 SE for Windows.

**Results:**

Eight studies involving 841 patients (841 eyes) were included in the meta-analysis. With a WMD of IOPR in the diurnal mean of 0.16 mmHg (95% CI, -0.31 to 0.63), the FCLT was as effective as FCDT in lowering IOP in patients with elevated IOP (P = 0.51). The WMDs of IOPR were 0.58 mmHg (95% CI: -0.002 to 1.17) at 8 AM, -0.07 mmHg (95% CI: -0.50 to 0.36) at 12 PM, and 0.41 mmHg (95% CI: -0.18 to 1.00) at 4 PM, and there were no signiﬁcant difference between FCLT and FCDT. FCLT was associated with a significantly lower incidence of eye pain, bitter taste, and irritation/stinging than FCDT, with pooled RRs of 0.34 (95% CI: 0.14 to 0.82), 0.06 (95% CI:0.008 to 0.42), and 0.35 (95% CI: 0.14 to 0.85), respectively.

**Conclusion:**

FCLT was associated with equivalent efficacy in IOP lowering comparing with FCDT. However, FCLT was better tolerated than FCDT.

## Introduction

Glaucoma, which causes optic nerve damage and loss of the visual field, is one of the most common causes of irreversible blindness worldwide[1]. It has been estimated that, in 2010, there were approximately 60 million glaucoma patients worldwide, and this figure is expected to rise to 80 million by 2020[2]. Elevated intraocular pressure (IOP) is recognized as the most important risk factor contributing to the development and progression of glaucoma. Glaucoma treatment is aimed at lowering IOP to preserve visual field and vision[3]. The Early Manifest Glaucoma Trial showed that IOP-lowering treatment decreased the risk of glaucoma progression by half[4]. Moreover, progression risk decreased about 10% with each millimeter of mercury (mmHg) of IOP reduction from baseline. In the Advanced Glaucoma Intervention Study, glaucoma patients with an IOP consistently <18 mm Hg had no discernible additional loss in the visual field over a six-year follow-up period[5]. Topical medical therapy is the mainstay of glaucoma treatment, and many topical medications are available for lowering IOP. While monotherapy with a single class of medication may be effective in lowering IOP, many patients require more two or more medications to reduce IOP to target levels[6]. Disadvantages of the multi-therapy approach include the washout effect, inconvenience, and poor adherence. 

More recently, several fixed combinations (FCs) combining two hypotensive agents in a single bottle have been developed. Their use may enhance adherence and tolerability together with a reduced exposure to preservatives such as benzalkonium chloride, which is known to exert toxic effects on the ocular surface. Moreover, FCs simplify dosing regimens and eliminate the washout effect[7,8]. 

Fixed combinations of 0.005% latanoprost/0.5% timolol (FCLT) and dorzolamide 2%/timolol 0.5% (FCDT) are both frequently used agents in primary open-angle glaucoma (POAG). Many previous studies have compared the efficacy and safety of FCLT with FCDT[9-18]. Some reported that FCLT was more effective in lowering IOP than FCDT[9-11,18], whereas others reported that two drugs had similar efficacy[12-17]. Such conflicting outcomes cannot afford us an exact guideline in clinical practice. Therefore, the present meta-analysis of randomized controlled trials of FCLT versus FCDT was undertaken to assess the efficacy and tolerability of the two drugs in the treatment of elevated IOP.

## Materials and Methods

This study was conceived, conducted, and reported according to the Preferred Reporting Items for Systematic Reviews and Meta-Analyses (PRISMA) statement for improving the quality of reports of randomized clinical trial (RCT) meta-analyses (Table S1) [19]. 

### 1: Search strategy

Randomized clinical trials were identiﬁed through a systematic search of PubMed, the ISI Web of Science, EMBASE, the Chinese Biomedicine Database, and the Cochrane Library (up to August 2013). The search combined terms related to prostaglandin analogs (including a MeSH search using the expressions “prostaglandins f, synthetic” and a keyword search using the words “latanoprost,” “Xalatan,” and “prostaglandin”), terms related to dorzolamide (including a MeSH search using the expressions “sulfonamides” and “thiophenes” and a keyword search using the words “dorzolamide” and “carbonic anhydrase inhibitor”), and terms related to timolol (including a MeSH search using the expressions “adrenergic beta antagonists” and “timolol” and a keyword search using the words “timolol,” “beta-blocker,” “beta blocker,” “β-blocker,” and “β blocker”). The search was limited to English and Chinese language papers and human subject studies. The reference lists of original reports and review articles retrieved through the search were reviewed for additional studies not yet included in the computerized databases. The Internet was searched using websites of professional associations and the Google Scholar search engine. Manufacturers of relevant pharmaceutical agents were also contacted for additional materials. 

### 2: Inclusion criteria

Published trials meeting the following criteria were incorporated into this meta-analysis: (1) study design: randomized clinical trials; (2) population: patients with elevated IOP; (3) intervention: FCLT versus FCDT after a washout period or runin period; (4) outcome variables: IOP reduction (IOPR) and adverse events; (5) publication parameters: written in English or Chinese; (6) duration: follow-up time of not less than one month. Meeting abstracts with insufﬁcient data, duplicate publications, letters, and reviews were excluded.

Two reviewers (H.M. and W.W.) determined the trial eligibility independently. First, the titles and abstracts of the obtained publications were screened. Then, the full articles of the remaining identified publications were scrutinized. Only trials meeting the inclusion criteria were assessed for methodological quality.

### 3: Outcome measures

The outcome measure of efficacy was the IOPR, representing the fluctuation from baseline to the end of treatment in IOP at 8 AM, 12 PM, and 4 PM as well as the diurnal mean IOP. Baseline was defined as time point after a washout period when patients used anti-glaucoma drugs or at the time of diagnosis. Diurnal mean IOP was defined as the average mean outcomes of the assessed day. When authors reported the mean and standard deviation (SD) of the IOPR, we used these directly. For studies that only reported absolute values for the IOP at baseline and the end point, IOPR and the SD of the IOPR (SD_IOPR_) were calculated as follows: IOPR = IOP_baseline_ - IOP_end-point_, SD_IOPR_ = (SD_baseline_^2^ + SD_end-point_^2^ - SD_baseline_ ×SD_end-point_)^1/2^[20]. We assessed tolerability by considering the proportions of patients with adverse events, including eye pain, bitter taste, irritation/stinging, superﬁcial punctate keratitis, vision reduction, watering, conjunctival hyperemia, itchiness, systemic disorders, and foreign-body sensation.

### 4: Data extraction

The data were extracted independently by two reviewers (H.M. and W.W.) according to a customized form that was used to record the names of the authors of the study, the year of publication, information on the study design, interventions, the location of the trial, the length of study, the number of subjects, patient age, sex, type of glaucoma, and IOP measurements. Furthermore, the number of withdrawals and patients reporting adverse events was also recorded. Any disagreement was resolved by discussion. If there were multiple reports for a particular study, data from the most recent publication were extracted.

### 5: Assessment of methodology quality

The methodological quality of each study was assessed using the r**i**sk-of-bias assessment tool outlined in the Cochrane Handbook for Systematic Reviews of Interventions (version 5.1.0)[21]. Two authors (H.M. and W.W.) subjectively reviewed all studies and assigned a value of “yes,” “no,” or “unclear” to the following: a) random sequence generation, b) allocation concealment, c) blinding (patients, personnel, and assessor), d) adequate assessment of each outcome, e) selective outcome reporting avoided, and f) no other bias.

### 6: Statistical analysis

The outcome measures were assessed on an ITT basis, the ITT population comprising all randomized patients who received study medication and provided a valid baseline measurement. Since the study groups usually could not be the same on all clinical characteristics, and variation existed in sample size, it was assumed that heterogeneity was present even when no statistical significance was identified, and it was decided to combine data using a random-effects model[22]. For dichotomous outcomes, the relative risk (RR) was estimated. The weighted mean difference (WMD) was calculated for continuous outcomes. All measures were estimated from each study with the associated 95% conﬁdence intervals (CIs) and pooled across studies. Statistical heterogeneity among studies was assessed with the χ^2^ and I^2^ tests. An I^2^ value greater than 50% indicates significant heterogeneity[23]. The overall effect was determined to be statistically significant with P < 0.05. The analysis was conducted using the Stata software package (Version 12.0; Stata Corp., College Station, TX).

### 7: Sensitivity analysis and publication bias

The sensitivity analysis was performed by computing the pooled results that excluded each study individually from the set of studies and comparing them with the pooled result from the set of studies. Potential publication bias was assessed visually with a funnel plot and statistically with the Egger and Begg tests[24,25].

## Results

### 1: Literature search

**Figure 1:**
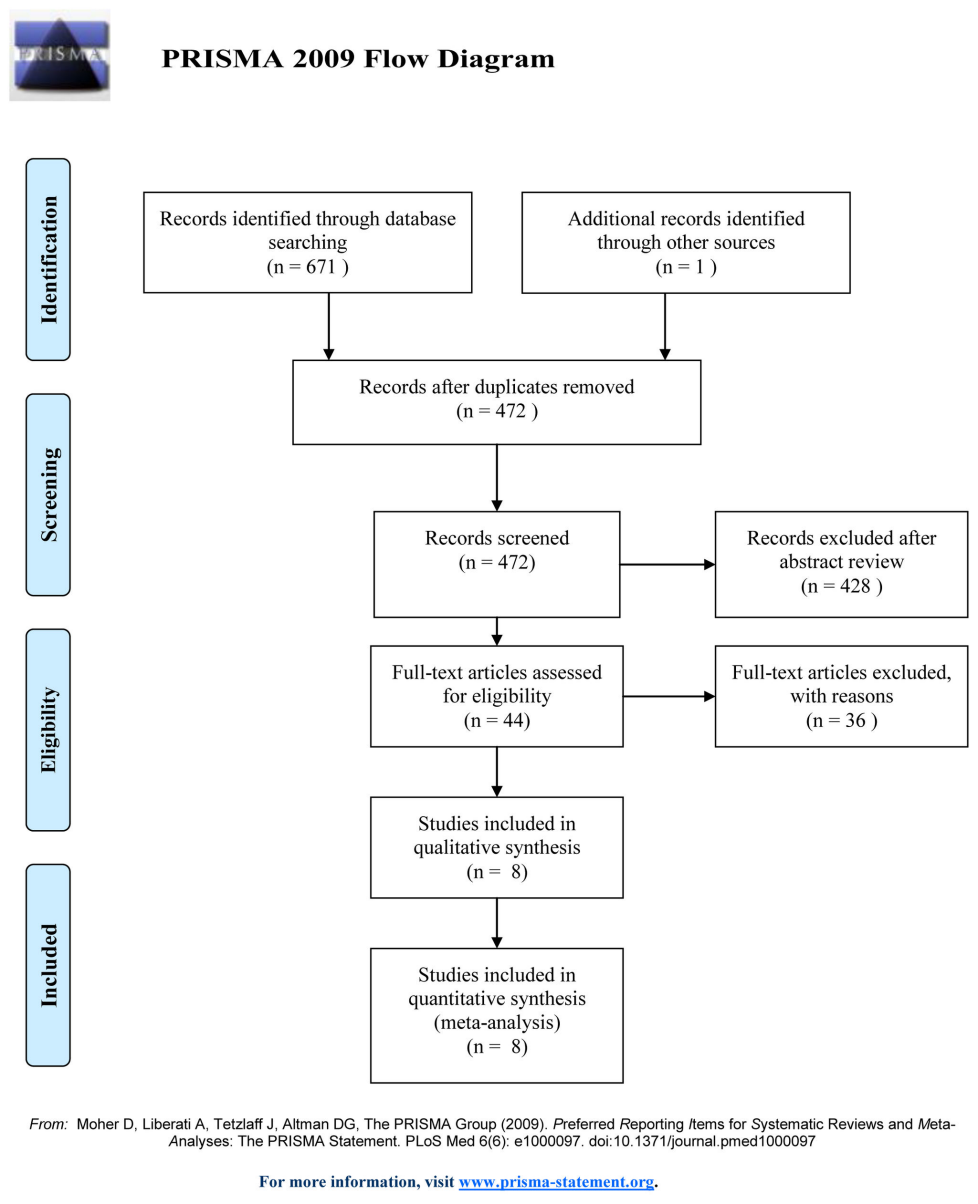
Process of study selection of RCTs.

The literature search identified 672 papers. Based on the content of the abstracts, 628 articles were found to be obviously ineligible for inclusion. The remaining 44 full-text articles were reviewed for a more detailed evaluation; 36 of them were also excluded because 10 compared FCLT with other fixed-combinations (FCLT versus travoprost/timolol fixed combination or FCLT versus bimatoprost/timolol fixed combination), 11 were not randomized (observational or non-randomized comparative studies), and one study did not report outcomes of interest (In adequate data on IOP or complications). Finally, eight RCTs[9,11,13-18] that met our inclusion criteria were included in the present meta-analysis. The flow of the studies included in our analysis is shown in Figure 1.

### 2: Characteristics of eligible studies

The main characteristics of the eight RCTs included in the meta-analysis are presented in Table 1. The studies were published between 2004 and 2012. In total, 841 eyes from 841 patients were evaluated (329 males, 476 females). Four hundred twenty eyes were included in the FCLT group, and 421 eyes were included in the FCDT group. The mean age ranged from 58.2 to 69.1 years. Three trials had a prospective, parallel design, and others had a prospective, crossover design. The duration of follow-up ranged from three to 12 months. The trials included were undertaken in various countries, including the U.S.A., Lithuania, Greece, Spain, Slovenia, and Turkey, and one study was conducted in multicenter in Europe.

**Table 1:** Characteristics of RCTs (n=8) included in the meta-analysis.

Trial(year)	Intervention	Eyes	Patients	Follow-up	Age	Sex(M/F)	Type of diagnostic			Withdrawal	Route
							POAG	OHT	Others		
Shin(2004)	FCLT	125	125	3m	64±11	58/67	86	37	2	4	8am
	FCDT	126	126	3m	63±12	54/72	72	50	4	7	8am,8pm
Miglior(2010)	FCLT	135	135	3m	65.8±11.3	67/68	92	32	11	1	evening
	FCDT	135	135	3m	66.6±10.0	54/81	100	23	12	0	morning,evening
Januleviciene(2009)	FCLT	15	15	12m	58.31±8.6	5/25	15	0	0	0	morning
	FCDT	15	15	12m	58.31±8.6	5/25	15	0	0	0	morning,bedtime
Konstas(2004)	FCLT	33	33	2m	64.57±12.7	13/20	25	5	3	1	8am
	FCDT	33	33	2m	64.57±12.7	13/20	25	5	3	1	8am,8pm
Martinez(2007)	FCLT	16	16	1m	68.7±7.3	9/7	10	0	6	-	8am
	FCDT	16	16	1m	69.1±6.4	8/8	9	0	7	-	8am 8pm
Konstas(2008)	FCLT	31	31	3m	62.8±12.2	16/15	18	0	13	3	8am
	FCDT	31	31	3m	62.8±12.2	16/15	18	0	13	3	8am 8pm
Cvenkel(2008)	FCLT	32	32	6w	61±12.7	7/25	32	0	0	0	8am
	FCDT	32	32	6w	61±12.7	7/25	32	0	0	0	8am 8pm
Eren(2012)	FCLT	33	33	6w	58.24±8.7	19/14	33	0	0	1	8am
	FCDT	33	33	6w	58.24±8.7	19/14	33	0	0	1	8am 8pm

POAG, primary open angle glaucoma; OH, ocular hypertension; IOPR, intraocular pressure reduction; FCLT, fixed-combination latanoprost 0.005%/ timolol 0.5%; FCDT, fixed-combination dorzolamide2.0%/timolol0.5%;M, male; F, female.

### 3: Quality assessment

The methodological quality of the RCTs included is presented in Table 2. The sequence generation was appropriate in two studies, while the others were unclear. Concerning selection bias, the risk is largely unclear because none of the studies provided information on the procedures for allocation concealment. The patients were blinded in four studies, personnel in four studies, and assessors in three studies. The adequate assessment of each outcome and selective outcome reporting avoided were all reported in the RCTs There were three double-blind studies and five single-blind studies. One trial was a multicenter RCT. Intention-to-treat analysis was used in five trials. All studies reported withdraws or dropouts. 

**Table 2:** Evaluation of the quality of RCTs included in the meta-analysis.

Author	Sequence Generation	Allocation Concealment	Blinding			Adequate assessment of each outcome	Selective reporting avoided	No Other Bias
			Patient	Personnel	Assessor			
Shin(2004)	unclear	unclear	no	no	yes	yes	yes	yes
Miglior(2010)	unclear	unclear	no	no	yes	yes	yes	yes
Januleviciene(2009)	unclear	unclear	yes	yes	no	yes	yes	yes
Konstas(2004)	unclear	unclear	yes	yes	no	no	yes	yes
Martinez(2007)	yes	unclear	no	yes	no	yes	yes	yes
Konstas(2008)	yes	unclear	no	no	yes	no	yes	yes
Cvenkel(2008)	unclear	unclear	yes	yes	no	yes	yes	yes
Eren(2012)	unclear	unclear	yes	no	no	no	yes	yes

### 4: Efficacy analysis

**Figure 2:**
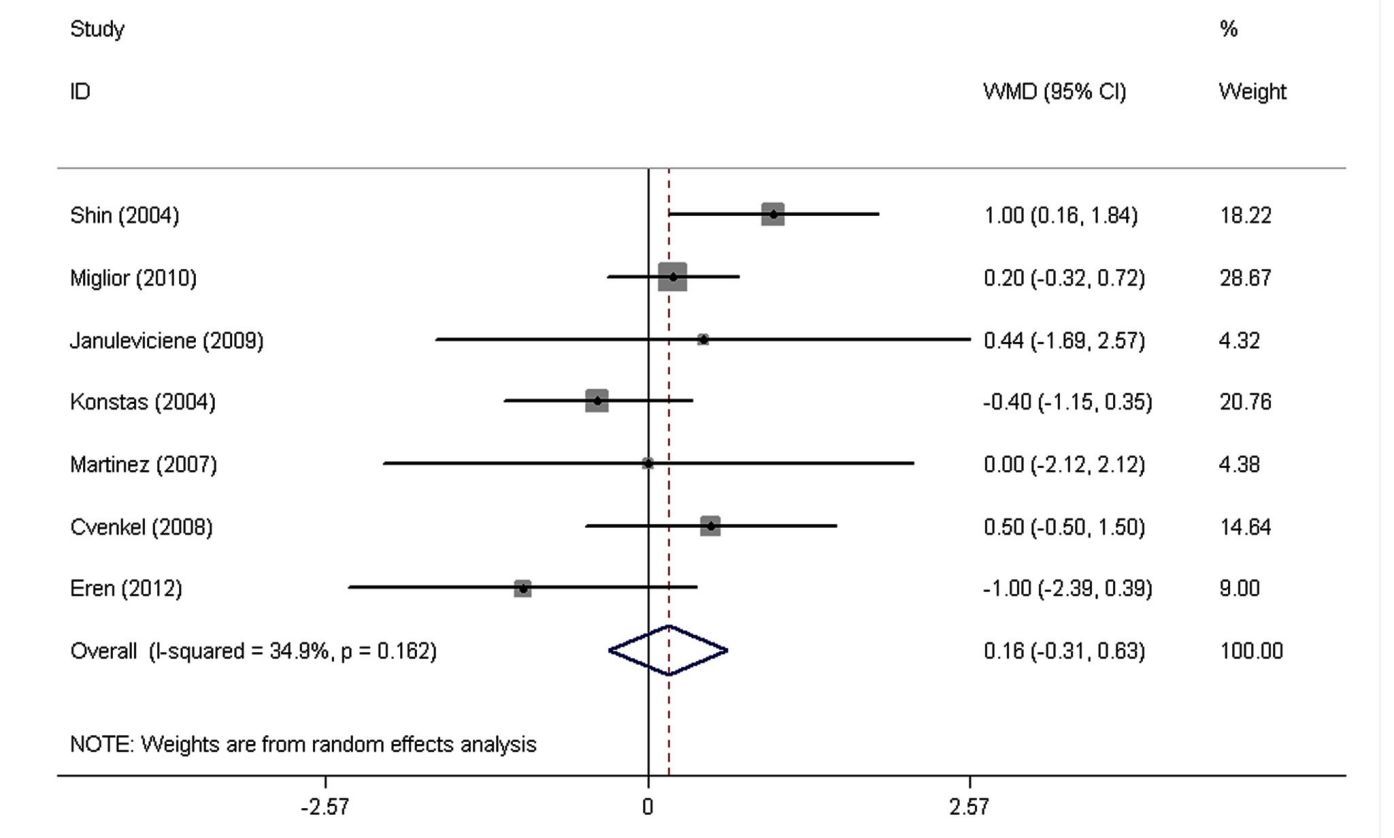
The weighted mean differences of the reduction in diurnal mean intraocular pressure between FCLT and FCDT . WMD indicates weighted mean difference, which were computed by using a random effects model. FCLT, fixed-combination latanoprost 0.005%/ timolol 0.5%; FCDT, fixed-combination dorzolamide2.0%/timolol0.5%.

**Figure 3:**
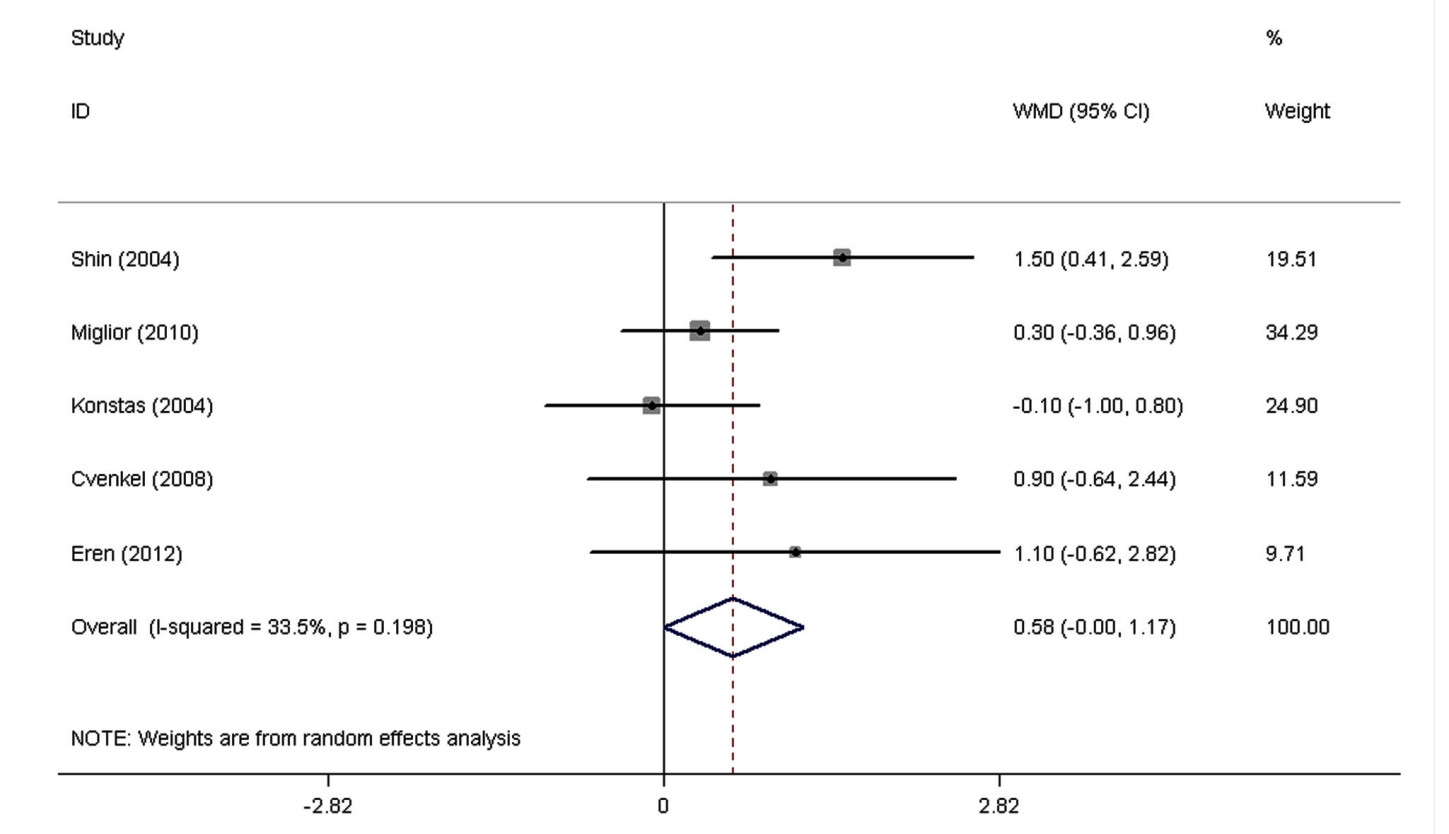
The weighted mean differences of the reduction in intraocular pressure between FCLT and FCDT at 8 AM. WMD indicates weighted mean difference, which were computed by using a random effects model. FCLT, fixed-combination latanoprost 0.005%/ timolol 0.5%; FCDT, fixed-combination dorzolamide2.0%/timolol0.5%.

**Figure 4:**
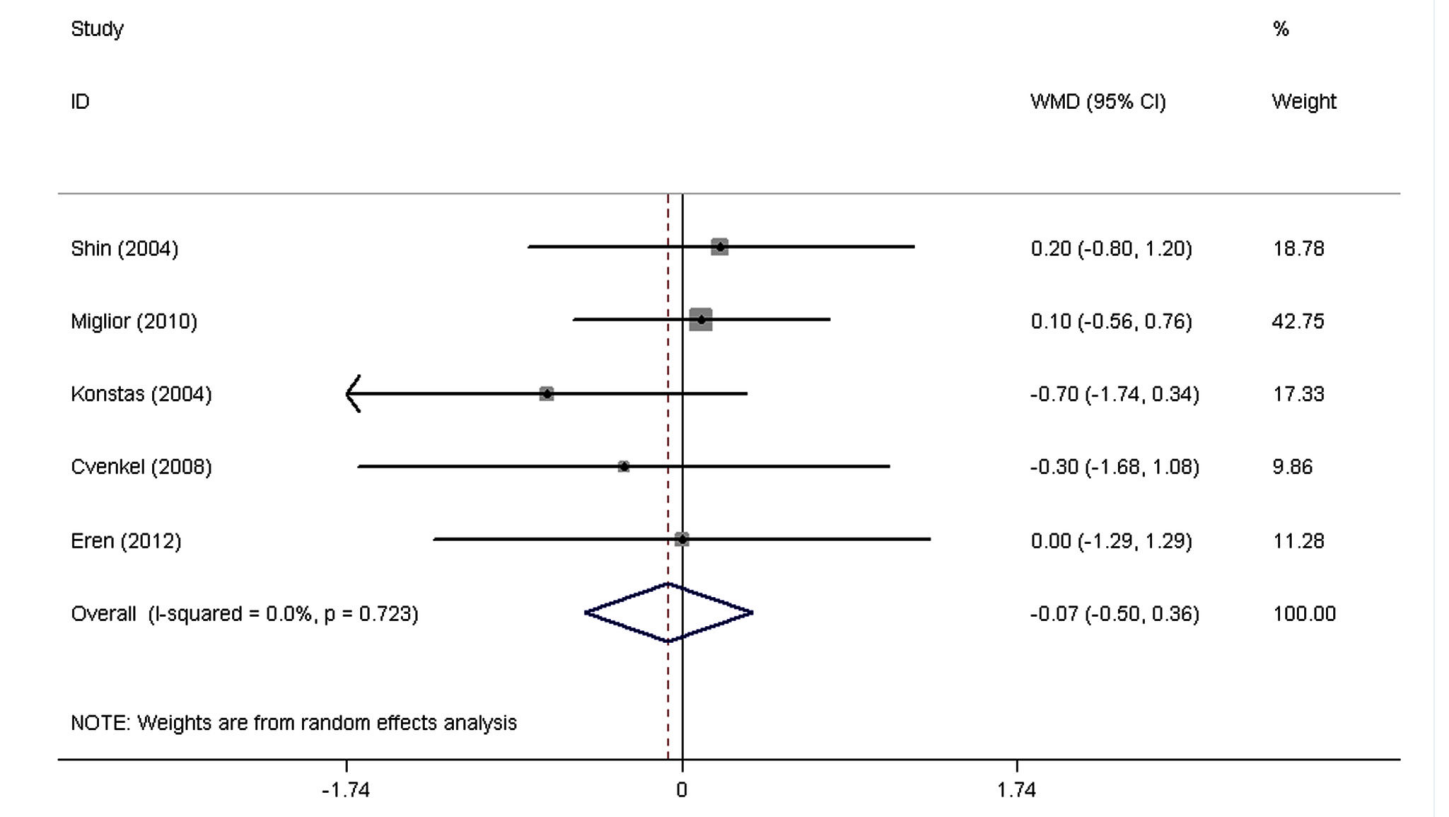
The weighted mean differences of the reduction in intraocular pressure between FCLT and FCDT at 12 PM. WMD indicates weighted mean difference, which were computed by using a random effects model. FCLT, fixed-combination latanoprost 0.005%/ timolol 0.5%; FCDT, fixed-combination dorzolamide2.0%/timolol0.5%.

**Figure 5:**
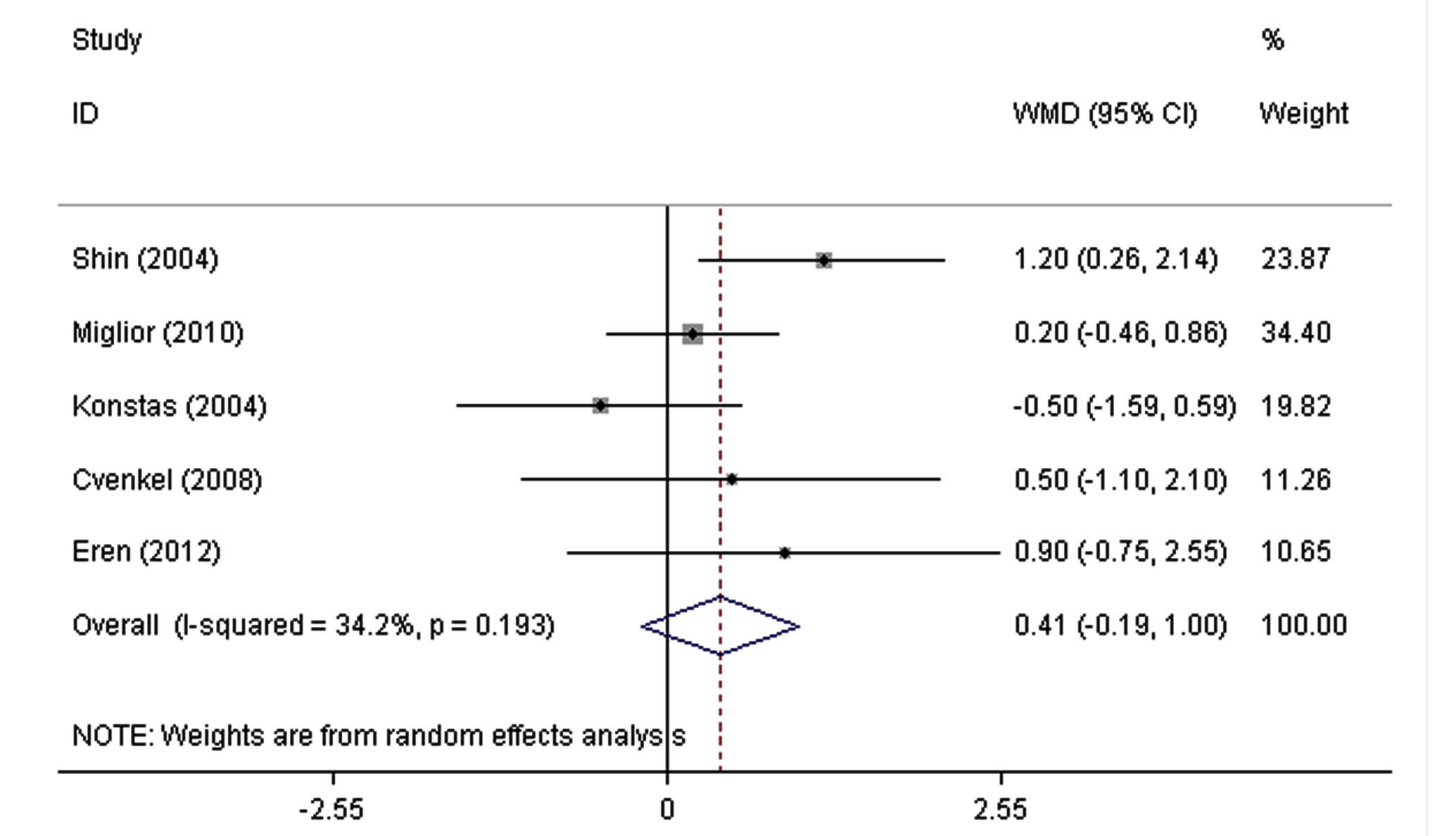
The weighted mean differences of the reduction in intraocular pressure between FCLT and FCDT at 4 PM. WMD indicates weighted mean difference, which were computed by using a random effects model. FCLT, fixed-combination latanoprost 0.005%/ timolol 0.5%; FCDT, fixed-combination dorzolamide2.0%/timolol0.5%.

The pooled WMDs of the IOPR comparison between FCLT and FCDT extracted from eight RCTs are shown in Figure 2-5. Both drugs significantly decreased IOP. No significant heterogeneity was found in the diurnal mean IOPR (P = 0.61, I^2=^34.9%), IOPR at 8 AM (P = 0.20, I^2=^33.5%), IOPR at 12 PM (P = 0.72, I^2=^0.0%), and IOPR at 4 PM (P = 0.19, I^2=^34.2%). With a WMD of IOPR in the diurnal mean of 0.16 mmHg (95% CI: -0.31 to 0.63), the FCLT was as effective as FCDT in lowering IOP in patients with elevated IOP (P = 0.51). The WMDs of IOPR were 0.58 mmHg (95% CI: -0.002 to 1.17) at 8 AM, -0.07 mmHg (95% CI: -0.50 to 0.36) at 12 PM, and 0.41 mmHg (95% CI: -0.18 to 1.00) at 4 PM, and there was no signiﬁcant difference between FCLT and FCDT.

### 5: Tolerability analysis

Incidences of eye pain [pooled RR: 0.340 (95% CI: 0.14 to 0.82)], bitter taste [pooled RR: 0.058 (95% CI: 0.008 to 0.42)], and irritation/stinging [pooled RR: 0.350 (95% CI: 0.14 to 0.85)] were significantly lower with FCLT than with FCDT (Table 3). There were no signiﬁcant differences between FCBT and FCDT in the incidence of any other reported adverse events, such as superﬁcial punctate keratitis, vision reduction, watering, conjunctival hyperemia, itchiness, systemic disorders, and foreign-body sensation.

**Table 3:** Rates of adverse events compared FCLT with FCDT.

Adverse events	Studies(n)	Crude event rate(n/N)		RR (95%CI)	Heterogeneity			Overall Effect	
		FCLT	FCDT		*I* ^*2*^(%)	*P*		*Z*	*P*
Eye pain	3	6/291	20/292	0.34 (0.14,0.82 )	0.00	0.558		2.40	0.016
Bitter taste	2	0/66	17/66	0.06 (0.01,0.42)	0.00	0.779		2.81	0.005
Irritation/stinging	3	0/97	20/97	0.35 (0.14, 0.85)	0.00	0.373		2.32	0.020
Superﬁcial punctate keratitis	3	3/97	6/97	0.59 (0.16, 2.21)	0.00	0.665		0.79	0.430
Vision reduction	5	5/366	8/366	0.80 (0.08 ,7.63)	57.60	0.069		0.19	0.846
Watering	2	6/64	1/64	4.31 (0.76, 24.53)	0.00	0.905		1.65	0.100
Conjunctival hyperemia	3	8/199	1/199	4.04 ( 0.85,19.18 )	0.00	0.736		1.76	0.079
Itchiness	2	6/166	0/166	7.00 (0.88, 55.89)	0.00	1.000		1.84	0.066
Systemic disorders	3	2/292	9/293	0.31 (0.09, 1.10)	0.00	0.957		1.82	0.069
Foreign-body sensation	1	1/31	0/31	3.00 (0.13, 70.92)	-	-		0.68	0.496

N: number of patients; n: number of patients with adverse events; RR: relative risk; 95% CI: 95% confidence interval; FCLT, fixed-combination latanoprost 0.005%/ timolol 0.5%; FCDT, fixed-combination dorzolamide2.0%/timolol0.5%.

### 6: Sensitivity analysis and publication bias

To assess the influence of each individual clinical trial included in the meta-analysis, one study was excluded at each time and the analysis performed again to determine the change in the IOPR. These exclusions did not alter the results obtained in previous analyses (data not shown). Funnel plots for the studies comparing FCLT with FCDT on the diurnal mean IOPR and IOPT at 8 AM, 12 PM, and 4 PM are qualitatively symmetrical, which indicated the absence of publication bias (data not shown). Begg’s and Egger’s tests confirmed these results.

## Discussion

Glaucoma aggravated by the progression of ocular hypertension could be prevented by IOP reduction. Medical therapy remains the cornerstone of glaucoma treatment[3]. Hence, it is important to select the simplest treatment regimen that achieves the most effective IOP reduction. A fixed combination has offered one more choice for ophthalmologists[26-28]. 

In the present meta-analysis, we reviewed eight randomized clinical studies comparing FCLT with FCDT in patients with elevated intraocular pressure. Both contained timolol, a beta blocker. In assessing the IOP, our study found that FCLT was associated with IOP-lowering efficacy comparable to that of FCDT, with a numerically higher but non-significant reduction in the IOP at 8 AM, 4 PM, and the diurnal mean, which is consistent with the results of an earlier review. However, FCLT was better tolerated than FCDT, with a significantly lower frequency of eye pain, bitter taste, and irritation/stinging. It is well known that PGAs give more conjunctival redness than other topical drugs (although this effect is diminished when a PGA is combined in a FC). In this study the conjunctival redness was not significantly more present in the FCLT. Further studies are warranted to confirm this result. 

Previous studies have shown that the addition of dorzolamide or latanoprost further lowers IOP in eyes on timolol, and latanoprost was as effective in lowering peak IOP as dorzolamide[29,30]. However, the efficacy of drugs used as adjunctive therapy may or may not mirror their efficacy when used in combination. One disadvantage of the FCLT is that one withdraws once daily timolol when switching from timolol twice daily to FCLT once daily.

In a previous meta-analysis, Cheng et al.[31] evaluated the IOP-lowering effect of the six most commonly used fixed-combination drugs containing 0.5% timolol. This work showed that LTFC was more effective than DTFC at the mean diurnal, peak, and trough IOPR. Some speciﬁc points may explain the discrepant ﬁndings, which are considered weak points in the former analysis. They summarized data by treatment group across trials, ignoring the randomized nature of trials and therefore resulting in non-randomized comparisons[32]. In addition, the authors reported the percentage change in IOP from baseline as the measure of effect, and they used the wrong formulae to calculate the variance of the change, which could have influenced the study results[32]. In contrast to these analyses, the present meta-analysis examined eight randomized clinical trials, used a wider range of clinical outcome measures, and focused on direct comparisons between FCLT and FCDT rather than making indirect inferences. The results suggest that FCLT has similar IOP-lowering efficacy compared to that of FCDT.

Our study provides additional interesting clues that may be useful for future research on the topic. Januleviciene et al.[13] compared the effects of FCLT versus FCDT on intraocular pressure, visual function, and retrobulbar blood flow in patients with open-angle glaucoma; these authors found no difference in visual field progression between DTFC and LTFC. In a longer non-randomized trial by Pajic et al.[12], though similar IOP-lowering effects were found in both treatment groups, FCDT seemed to be more effective in preventing glaucomatous visual field progression. These findings may be explained by the fact that dorzolamide significantly improves ocular hemodynamic parameters, and there is increasing evidence that reduced ocular blood flow is directly associated with visual field loss[33-36]. Few published studies have investigated the long-term efficacy and prognosis comparing FCLT to FCDT. Thus, one may focus on these outcomes to better address mechanical differences underlining FCLT and FCDT. More large-scale and well-performed RCTs are warranted.

The strengths of the current meta-analysis are as follows. First, as far as we know, our research is the first classical pairwise meta-analysis (direct comparisons) comparing the efficacy and safety of FCLT and FCDT[37,38]. Second, a relatively high number of the included studies provided a significant degree of power for the analysis. Third, the likelihood of bias was minimized by developing a detailed protocol before initiating the study, by performing a meticulous search for published studies, and by using explicit methods for study selection and data extraction. Fourth, the quality assessment was conducted according to the Cochrane Handbook for Systematic Reviews of Interventions. Furthermore, we used the random effect model, which is a relatively conservative statistical analysis method. Moreover, the sensitivity analysis demonstrated that the conclusions from this analysis were robust because the overall outcomes remained the same when any clinical trail was excluded. Finally, funnel plots were created to detect potential publication biases, and the Begg and Egger tests indicated a low possibility of publication biases.

Despite these advantages, some limitations of the current study should not be ignored. First, the characteristics of populations and differences in dosage, route, timing, and duration of administration may result in heterogeneity and have a potential impact on our results. However, no statistically heterogeneity was found in this meta-analysis for all outcomes. Second, our analyses of the IOPR and adverse events were based on data pooled from trials of different durations; owing to the lack of data reported in all phases of follow-up, we developed a compromise proposal to choose the data from the follow-up endpoint. It should also be noted that it is likely that some useful articles were missed, especially those published in other languages, although multiple databases and websites were searched[10]. Finally, none of the identified RCTs provided a cost-effectiveness analysis; thus, this may be an interesting focus for future studies. 

The results of our meta-analysis including eight randomized clinical trials suggest that FCLT provides similar IOP-lowering efficacy to that of FCDT. FCLT seems to be better-tolerated than FCDT. Therefore, FCLT may be a better choice for patients with elevated IOP. Pragmatic randomized controlled trials lasting longer and with broader population inferences are needed to confirm our results further. 

## Supporting Information

Table S1 **PRISMA checklist.**
(DOC)
